# Principal Components and Cluster Analysis of Trace Elements in Buckwheat Flour

**DOI:** 10.3390/foods12010225

**Published:** 2023-01-03

**Authors:** Mengyu Zhao, Junbo Gou, Kaixuan Zhang, Jingjun Ruan

**Affiliations:** 1 College of Agronomy, Guizhou University, Guiyang 550025, China; 2 Pharmacy Faculty, Hubei University of Chinese Medicine, Wuhan 430065, China; 3 Institute of Crop Science, Chinese Academy of Agriculture Science, Beijing 100081, China

**Keywords:** buckwheat flour, trace elements, principal component analysis, cluster analysis, dietary nutrition

## Abstract

Essential trace elements are required at very low quantities in the human body but are essential for various physiological functions. Each trace element has a specific role and a lack of these elements can easily cause a threat to health and can be potentially fatal. In this study, inductively coupled plasma mass spectrometry (ICP-MS) and inductively coupled plasma atomic emission spectrometry (ICP-AES) were used to determine the content of trace metal elements Ca, Fe, Cu, Mg, Zn, Se, Mo, Mn, and Cd in buckwheat flour. The content and distribution characteristics of trace metal elements were investigated using principal component and cluster analysis. The principal component analysis yielded a four-factor model that explained 73.64% of the test data; the cumulative contribution of the variance of the 1st and 2nd principal factors amounted to 44.41% and showed that Cu, Mg, Mo, and Cd are the characteristic elements of buckwheat flour. The cluster analysis divided the 28 buckwheat samples into two groups, to some extent, reflecting the genuineness of buckwheat flour. Buckwheat flour is rich in essential trace metal elements and can be used as a source of dietary nutrients for Mg and Mo.

## 1. Introduction

Buckwheat belongs to the *Fagopyrum* genus of the *Polygonaceae* family. It can be divided into two groups according to the grain size, large and small [1,2,3]. Buckwheat is a traditional grain crop for both medicine and food and has high nutritional value and benefits [4,5,6]. The amount of protein, fat, vitamins, and metal elements in buckwheat are higher than those in rice, wheat, corn, and other staple foods. It is especially rich in rutin, vitamins, crude fiber, and trace elements which can reduce serum cholesterol and have anti-aging effects; preventing and treating heart disease, cerebrovascular disease, and diabetes [7,8]. Buckwheat is an important dietary resource of essential trace elements for the human body and is increasing in popularity [9,10]. Accurately exploring the distribution characteristics of metal elements in buckwheat resources is key to ensuring a thorough understanding of the authenticity and classification of buckwheat resources [11,12,13].

The basic factors that determine the content and distribution of metal elements in plants are their variety and other factors, such as origin, climate, and season. However, genetically similar plants have certain restrictions on the distribution and content of metal elements that are absorbed from the soil and accumulate in the plants. These patterns of distribution and content are widely used as the basis for the identification of the authenticity of some plants’ medicinal materials [14,15,16]. Chemometrics has been successfully applied in various fields and is a powerful tool for data analysis and processing, with principal component and cluster analysis being the most commonly used analytical methods [17,18,19,20]. This study used ICP-MS and ICP-MS to determine the content of Ca, Fe, Cu, Mg, Zn, Se, Mo, Mn, and Cd in buckwheat resources. The spectral data of buckwheat flour were studied using principal component and cluster analysis to identify the nutritional characteristics of buckwheat flour.

## 2. Materials and Methods

### 2.1. Materials, Reagents, and Instruments

See Supplementary Table S1 for the species and distribution of buckwheat samples. Standard solutions of Ca, Fe, Cu, Mg, Zn, Se, Mo, Mn, and Cd (diluted stepwise as required during use) were provided by the Beijing Nonferrous Metals Research Institute (Beijing, China). All standard solutions were provided by the National Nonferrous Metals and Electronic Materials Testing Center and National Standards Center. Nitric acid, perchloric acid, 10% NH_4_H_2_PO_4_ (10%), and 20 mg/L Pd (solution) were of excellent purity. Ultra-pure water (18.2 MΩ·cm) was purchased from Sigma-Aldrich (St Louis, MO, USA). Both 70% nitric acid and 30% hydrogen peroxide solutions were BV- III ultra-clean and high-purity.

The instruments and equipment used in the experiment were ICP-MS 7900 inductively coupled plasma mass spectrometer (Agilent Company, Palo Alto, CA, USA), ICP-OES 5900 inductively coupled plasma atomic emission spectrometer (Agilent Company, Palo Alto, CA, USA), a microwave digestion instrument (MASTER-40, Shanghai Xinyi Microwave Chemical Technology Co., Ltd., Shanghai, China), an acid catcher (TK12, Shanghai Xinyi Microwave Chemical Technology Co., Ltd., Shanghai, China), and an electronic balance (Sartorius, Göttingen, Germany).

### 2.2. Sample Handling

#### 2.2.1. Sample Pretreatment

The glassware used in the experiment was first washed with water, then soaked in a 4.6657 mol/mL 30% nitric acid solution for more than 24 h, washed with distilled water, and then washed with ultrapure water (18.2 MΩ·cm) [21]. Twenty-eight buckwheat samples (Supplementary Table S1) were collected and planted at the teaching experimental farm of Guizhou University for two consecutive years, from June to October 2020 and June to October 2021. The seeds were harvested in October 2021, the bran was removed, and the samples were dried at 80 °C for three days. All the samples were crushed and sieved through 80 mesh screens before being bottled for storage [22]. The mixed standard working solutions were prepared by stepwise dilution with HNO_3_ (2%) and Rhodium (Rh) was used as the internal standard element to obtain two concentration gradients of 0.1–100 μg/L and 0.01–10 μg/L, respectively.

#### 2.2.2. Sample Digestion

Microwave digestion technology was used to digest all buckwheat samples [23]. The sample was heated in an oven at 60 °C for 1 h and was manually shaken for 5 min. Accurately weighed 0.25 g (accurate to 0.0001 g) of buckwheat sample was put into a total fatty matter (TFM) digestion tank, 8 mL of concentrated nitric acid was added, heated for 1 h under 130 °C of an acid extractor, put into the outer tank, tighten the seal, and put it into a microwave digestion instrument for digestion. After the digestion procedure was completed and cooled, taken out of the digestion tank, opened, and placed in the acid remover at 130 °C for acid removal, until the solution was concentrated into a small drop of liquid residue, 5 mL of dilute nitric acid (2%, *v/v*) was added, and continued to be heated and dissolved it in the acid remover at 130 °C for 20 min so that the elements to be measured can be fully transferred to the solution. After the solution cooled down, the solution was transferred quantitatively to a 50 mL plastic volumetric flask, 0.5 mL of rhodium standard working solution (1.0 μg/mL) was added, and shaken well with diluted nitric acid (2%, *v/v*) to obtain the solution to be measured [24]. Under the same conditions, a sample control test was conducted. Three biological replicates were considered for each experiment.

### 2.3. Determination of Trace Elements

ICP-MS was used to determine the trace elements in buckwheat flour, while ICP-AES was used to determine the relatively high elements [25]. The determination of Ca, Fe, Cu, Mg, Zn, Mo, Mn, and Cd was performed using the method of Golijan et al. [26] and Çelik et al. [27]. The determination of Se was performed using the method of Jiang et al. [28]. Under the operating working conditions of the instrument (Table 1), it was confirmed that the sensitivity, oxide, double charge, and other indicators of the instrument meet the requirements, and the determination method was edited. We tested the blank solution, standard solution, and sample solution in turn. The instrument automatically draws the standard curve and calculates the content of each element in the sample according to the curve regression equation.

(1) $$\mathrm{Content}\;\mathrm{of}\;\mathrm{metal}\;\mathrm{elements}\;\mathrm{in}\;\mathrm{the}\;\mathrm{sample}\;(\mu g/\mathrm{mL})=\frac{C\times V\times f}{m}$$

where: *C* is the mass concentration of the sample solution (μg/mL), *V* is the constant volume of the sample solution (mL), *f* is the dilution factor of the sample solution, and *m* is the sample mass (g).

### 2.4. Data Processing

SPSS 23.0 statistical software (IBM Corp., Armonk, NY, USA) was used to analyze and process the data, and the original data were standardized for principal component analysis and cluster analysis.

## 3. Results

### 3.1. Determination of Nine Traces Mineral Elements in Buckwheat Flour

We detected nine trace elements in eight *Fagopyrum esculentum* Moench., fourteen *Fagopyrum tataricum* (L.) Gaertn., and six *Fagopyrum dibotrys* (D. Don) Hara. Supplementary Table 1 shows the content, variation range, average value, and standard deviation of the nine metal elements in the buckwheat seeds. The contents of Mg, Ca, Fe, Zn, Mn, and Cu are relatively high, while the contents of Se, Mo, and Cd are relatively low in twenty-eight buckwheat varieties. According to the average value of each element, the content order of the nine elements in 28 buckwheat seeds was: Mg > Ca > Fe > Zn > Mn > Cu > Se > Mo > Cd. The highest content is Mg, and the lowest is Cd.

### 3.2. Principal Component Analysis

**Figure 1:**
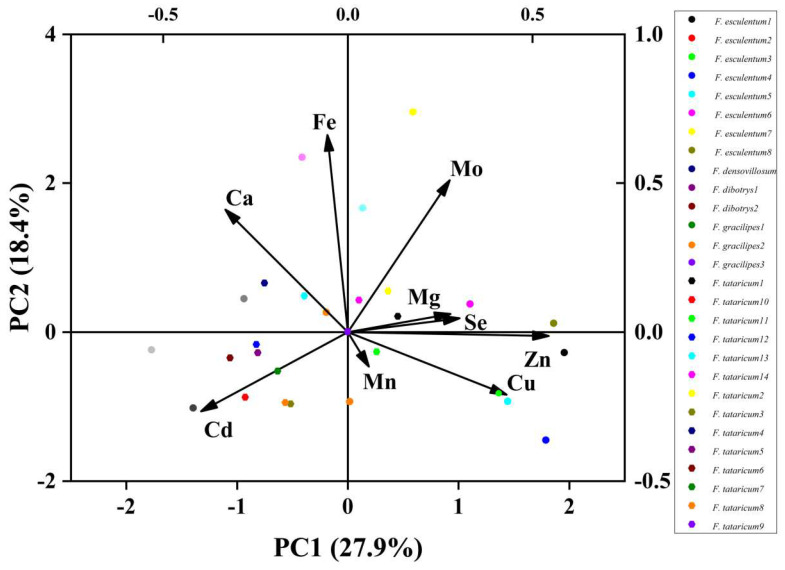
Principal component analysis of trace elements in buckwheat flour.

One of the purposes of principal component analysis is to describe the relationship between multiple indicators or factors using a small number of factors [29]. Principal component analysis has a potential requirement for the original variables to be strongly correlated [24]. In this study, a correlation analysis of the original variables was conducted during the principal component analysis, and the correlation coefficient matrix between the nine variables was calculated. The results show that nearly 50% of the correlation coefficients were greater than 0.3 and that all variables were linearly correlated with at least one other variable; indicating that these variables were suitable for principal component analysis. The eigenvalues and contribution rates of the principal components were the basis for the selection of the principal components. Figure 1 shows the overall description of the original variables by the initial solution of the principal component analysis, and the results after the factor load matrix were rotated by the maximum variance orthogonal method. Table 2 shows four factors contributed to 73.64% of the total variance; therefore, a four-factor model explains 73.64% of the data. Of the main factors, the first factor was highly positively correlated with Cu and Mg, the second was highly positively correlated with Mo and highly negatively correlated with Cd, the third was highly positively correlated with Fe and Ca, and the fourth was highly positively correlated with Mn. Because nearly 50% of the total variance is contributed by the first two main factors, it can be considered that Cu, Mg, Mo, Ca, Fe, and Mn are characteristic elements of buckwheat flour.

### 3.3. Cluster Analysis

**Figure 2:**
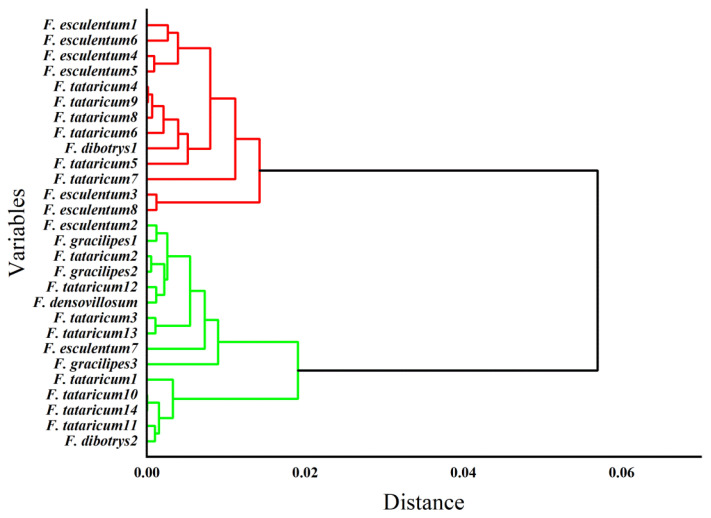
Dendrogram of cluster analysis for trace elements in buckwheat flour.

The Euclidean square sum distance was used as the cluster distance and Ward’s method was used for clustering [30]. Twenty-eight buckwheat varieties were divided into two categories. The *F. esculentum* in southwest China, the *F. tataricum* in Yunnan Guizhou Sichuan’s high altitude area, and the *F. dibotrys* in Sichuan high altitude area belong to a large group. The rest of the buckwheat belongs to another large group. The results show that the altitude has an effect on the content of trace elements in buckwheat, which is consistent with the research results of Pongrac et al. Figure 2 shows that when the distance was approximately 0.001, *F. esculentum* 3 and *F. esculentum* 8 are clustered together, indicating that they are closely related. They form a cluster together with *F. esculentum* 1, *F. esculentum* 6, *F. esculentum* 4, and *F. esculentum* 5, indicating the authenticity of buckwheat flour. *F. denisovillosum, F. gradients* 1, *F. gradients* 2, and *F. gracilipes* 3 cluster together, which shows that they not only have a close relationship but also reflect their authenticity. When the distance was approximately 0.06, 28 buckwheat samples are divided into two categories: Category 1 includes six *F. esculentum* samples; further, it also contains *F. dibotrys* 1 and, six *F. tataricum* samples respectively *F. tataricum* 4, *F. tataricum* 9, *F. tataricum* 8, *F. tataricum* 6, *F. tataricum* 5 and *F. tataricum* 7. Category 2 contains eight *F. tataricum* samples, one *F. dibotrys* 2, two *F. esculentum* samples, and four small grain groups of wild buckwheat. The clustering results indicated that there are significant genetic differences in trace metals among the different buckwheat varieties. There is a specific relationship between the chemical components of plants and their genetic relationships, and species with similar genetic relationships often contain the same chemical components. In addition, buckwheat flour shows authentic characteristics.

## 4. Discussion

Inductively coupled plasma mass spectrometry (ICP-MS) and inductively coupled plasma atomic emission spectrometry (ICP-AES) have the advantages of a low detection limit, wide linear range, high sensitivity, fast analysis speed, less sample consumption, low interference, and simultaneous determination of multiple elements [11,24]. In this study, ICP-MS was used to determine trace elements in buckwheat flour, while ICP-AES was used to determine relatively high elements. To reduce contamination and the loss of volatile elements during sample pretreatment, microwave digestion technology was used to digest all buckwheat samples. Using microwave digestion, ICP-MS, and ICP-AES, analysis, and determination of the trace element content information in buckwheat flour can be obtained quickly and accurately.

In 2009, Zhang et al. [24] studied the element concentration and dietary intake of Chinese food and recommended that an adult need to ingest the following elements from food every day: Fe 5.4~46.7 mg/d; Mn 1.1~8.8 mg/d; Zn 4.09~22.3 mg/d; Cu 0.45~5.8 mg/d; Ca 222~1660 mg/d; Mg 120~680 mg/d; Mo 52~523 μg/d; and Se 8~272 μg/d. The results of our study show that buckwheat is rich in Ca, Fe, Cu, Mg, Zn, Se, Mo, Mn, and Cd, and can meet the dietary requirement of various elements. It has been reported that buckwheat could be used as a dietary nutrient source for Zn, Cu, Mn, and Se [11,30]. According to the dimension reduction results of the principal component analysis, our study concluded that Cu, Zn, Mo, Fe, and Ca are characteristic elements in buckwheat, which failed to fully support the conclusion from previously published [24,31,32] (Figure 1). From the abundance and characteristics of each element in buckwheat, we believe that buckwheat can also be used as a dietary source of the essential macro element Mg and non-essential element Mo for the human body.

Ca, Fe, Mn, Zn, Mg, Mo, Cu, and other essential elements of the human body participate in the normal physiological and biochemical processes of the human body by forming chelates with nucleic acids, proteins, enzymes, hormones, and other biological organic substances; and play a role in maintaining the normal functions. Buckwheat flour is also rich in Zn, which is an essential trace element in the body. Zn regulates immune function and regulates and controls the metabolism through the enzyme system to achieve antibacterial and antiviral effects. Borovaya et al. [33] found that the accumulation of Zn in the soil can increase the rutin content in buckwheat grains. The content, type, and aeration of mineral elements in soil will affect the content of trace elements in buckwheat flour. Ozyazici et al. [34] found that the use of earthworms in the soil can increase the mineral content of buckwheat. Buckwheat processing technology also has a significant impact on the mineral content and bioavailability of buckwheat processed products [35]. Therefore, if we want to introduce buckwheat containing multiple mineral elements to the dining table and take advantage of its therapeutic value, we need to choose planting sites in rural areas, far away from the urban suburbs to eliminate the potential pollution from heavy metals.

## 5. Conclusions

The content of nine metal elements in eight *F. esculentum* Moench., fourteen *F. tataricum* (L.) Gaertn., and six *F. dibotrys* (D. Don) Hara flour samples were determined and indicated that buckwheat resources were rich in metal elements required by the human body. Principal component analysis showed that Cu, Mg, and Mowere are characteristic trace elements of buckwheat resources. The clustering results showed that some genetic differences in the distribution of the nine elements in the seeds of different buckwheat varieties. This study not only provided data on nine metal elements in 28 buckwheat resource seeds but also discussed the characteristics of metal elements in buckwheat resources using chemometrics, providing a theoretical basis for the identification of dietary nutritional characteristics of buckwheat flour.

## Figures and Tables

**Table 1:** ICP-MS operating parameters.

Instrument Index	Working Conditions
Incident power/W	1300
Auxiliary air flow/(L·min^−1^)	1.3
Aperture of sampling cone/mm 1.0	1.0
Plasma gas flow/(L/min)	15.0
Vacuum degree of analysis chamber/Pa	4.54 × 10^−4^
Sample lifting rate/ (r ·s^−1^)	0.3
Dwell time/ms	10
Cooling gas flow/(L min^−1^)	13.0
Atomizing gas flow/(L min^−1^)	0.9
Aperture of intercepted cone/mm	0.6
Atomizing chamber temperature/**°C**	2
Measurement method	Jump peak
Analysis time/s	45
Resolution ratio/amu	0.7
Carrier gas flow (L/min)	1.0
Integration time of each element/ms	300

**Table 2:** Total variance description through principal component analysis and varimax orthogonal rotated factor loading matrix.

Trace Element Name	Principal Components			
	1	2	3	4
Mg	0.752	0.023	0.191	0.168
Ca	−0.257	−0.257	0.796	−0.028
Fe	0.145	0.252	0.873	0.028
Zn	0.647	0.136	−0.064	0.600
Mn	0.177	−0.171	0.081	0.881
Cu	0.838	0.082	−0.306	−0.046
Se	−0.211	0.536	−0.205	0.472
Mo	0.097	0.811	0.147	−0.136
Cd	−0.106	−0.775	0.059	0.001
Eigenvalue	2.39	1.60	1.57	1.04
Variance contribution	26.60	17.81	17.47	11.58
Cumulative variance contribution rate	26.60	44.41	61.88	73.64

Note: The underlined data indicates that the load factor value is greater than 0.70 or less than −0.70.

## Data Availability

Data supporting reported results are available upon request.

## Supplementary Materials

The following are available online at https://www.mdpi.com/article/10.3390/foods12010225/s1, Table S1. Name, Source and Trace Element Content of 28 Buckwheat Samples.
