# Supplementary material for: Principal Components and Cluster Analysis of Trace Elements in Buckwheat Flour
Source: Foods. 2023 Jan 3;12(1):225. doi: 10.3390/foods12010225 (PMC9818536; doi:10.3390/foods12010225)
Supplement: Supplementary file 1 [file foods-12-00225-s001.zip › foods-2113804-supplementary.pdf]

Table S1. Name, Source and Trace Element Content of 28 Buckwheat Samples.

Buckwheat No.	Collection Place	Mg (μg/g)	Ca (μg/g)	Fe (μg/g)	Zn (μg/g)	Mn (μg/g)	Cu (μg/g)	Se (μg/g)	Mo (μg/g)	Cd (μg/g)
F. esculentum 1	Aba Prefecture, Sichuan Province	248.8±24.9	177.49±17.6	62.23±6.2	39.08±3.9	10.08±1	9.98±1.01	1.41±0.14	0.35±0.03	0.03
F. esculentum 2	Xichang City, Sichuan Province	247.25±24.6	304.9±30.4	83.5±8.2	26.24±2.7	10.15±1.1	7.84±0.78	1.46±0.15	0.3±0.03	0.05
F. esculentum 3	Shuicheng County, Guizhou Province	249.38±25	120.54±12	67.16±6.6	36.09±3.6	10.29±1	10.4±1.05	0.34±0.03	0.33±0.03	0.03
F. esculentum 4	Weining County, Guizhou Province	249.72±26	144.37±14.5	55.91±5.6	35.21±3.51	10.15±1.1	12.76±1.2	0.72±0.07	0.31±0.03	0.03
F. esculentum 5	Zunyi City, Guizhou Province	248.92±24.5	154.61±15.5	59.03±5.8	39.3±3.9	10.32±1.1	9.61±0.96	0.76±0.07	0.32±0.03	0.03
F. esculentum 6	Xi'an City, Shanxi Province	244.78±24.3	159.85±16	70.99±6.98	30.54±3	9.36±0.9	8.51±0.84	0.9±0.08	0.37±0.04	0.02
F. esculentum 7	Xi'an City, Shanxi Province	246.76±24.4	320.93±32	112.78±11.3	33.14±3.4	9.67±0.96	7.44±0.75	0.75±0.08	0.4±0.04	0.02
F. esculentum 8	Jiujiang City, Jiangxi Province	248.2±25	132.57±13	65.68±6.6	35.84±3.6	10.09±1.08	8.83±0.87	1.30±0.1	0.39±0.04	0.04
F. tataricum1	Jiujiang City, Jiangxi Province	247.29±24.7	218.41±22	92.02±9.3	11.54±1.54	11.54±1.2	6.48±0.6	0.82±0.08	0.29±0.03	0.02
F.	Xi'an	249.15±2	274.52±2	81.69±8.2	9.53±0.9	9.53±0.9	7.29±0.7	0.77±0.	.39±0.0	0.0

tataricum2	City, Shanxi Province	5	7.5		6	6	2	08	4	2
F. tataricum3	Xi'an City, Shanxi Province	247.81±24.8	280.24±28	110.17±11	9.84±1.1	9.84±0.99	5.83±0.57	0.68±0.7	0.36±0.03	0.02
F. tataricum4	Weining County, Guizhou Province	247.01±24,7	168.2±17	70.15±6.96	9.68±0.9	9.68±0.96	6.32±0.63	0.41±0.04	0.31±0.03	0.18
F. tataricum5	Weining County, Guizhou Province	246.4±24.5	144.01±14.5	56.84±5.8	9.82±0.97	9.82±0.97	6.82±0.68	0.94±0.09	0.3±0.03	0.03
F. tataricum6	Weining County, Guizhou Province	245.94±24.6	164.62±16.5	82.83±8.4	9.76±0.9	9.76±0.96	8.49±0.86	0.51±0.05	0.35±0.03	0.03
F. tataricum7	Weining County, Guizhou Province	248.62±24.8	151±15	93.78±9.4	10.4±0.99	10.4±1.03	10.77±1.09	0.46±0.04	0.34±0.03	0.05
F. tataricum8	Weining County, Guizhou Province	236.02±23.6	158.9±16	57.51±5.7	9.57±.09	9.57±0.96	6.65±0.67	0.54±0.05	0.32±0.03	0.06
F. tataricum9	Zhaotong City, Yunnan Province	247.67±24.5	165.15±16.5	69.39±6.9	9.69±0.97	9.69±0.97	9.35±0.95	0.67±0.06	0.34±0.03	0.05
F. tataricum10	Zhaotong City, Yunnan Province	237.79±23.7	218.11±21.8	67.83±6.8	9.34±0.9	9.34±0.93	7.29±0.73	0.68±0.07	0.32±0.03	0.05
F. tataricum11	Xichang City, Sichuan Province	246.82±24.6	210.55±21	75.25±7.5	10.5±1.021	10.5±1.05	6.05±0.7	0.68±0.07	0.37±0.03	0.08
F. tataricum12	Guiyang City, Guizhou Province	237.3±23.6	284.41±28.5	59.03±5.9	10.04±0.9	10.04±1.02	4.36±0.46	0.52±0.05	0.32±0.03	0.09
F.	Shuiche	244.51±2	267.85±2	93.4±9.3	10.48±1.	10.48±1.	7.74±0.7	0.65±0.	0.32±0.	0.1

tataricum13	ng County, Guizhou Province	4.6	6.6		05	05	9	06	03	1
F. tataricum14	Zunyi City, Guizhou Province	245.77±24.5	225.32±22.4	68.72±6.9	10.53±1.1	10.53±1.05	8.88±0.9	0.77±0.07	0.29±0.03	0.1
F. gracilipes1	Xichang City, Sichuan Province	250.18±25	315.75±31.4	81.65±8.4	9.04±0.9	9.04±0.9	8.56±0.88	0.32±0.03	0.31±0.03	0.06
F. gracilipes2	Xichang City, Sichuan Province	248.13±24.7	288.94±29	82.83±8.3	9.96±0.98	9.96±0.99	9.46±0.97	0.63±0.06	0.28±0.03	0.11
F. gracilipes3	Ya'an City, Sichuan Province	249.57±24.8	343.98±34.5	64.13±6.3	10.62±1.1	10.62±1.08	7.67±0.78	0.28±0.02	0.29±0.03	0.08
F. densovillosum	Xichang City, Sichuan Province	249.59±25	276.43±27.9	64.14±6.4	9.78±0.97	9.78±0.97	8.21±0.83	0.97±0.1	0.31±0.03	0.13
F. dibotrys1	Xichang City, Sichuan Province	248.53±24.7	188.63±18.9	60.25±6	9.64±0.9	9.64±0.96	6.67±0.8	0.63±0.06	0.28±0.03	0.06
F. dibotrys2	Ya'an City, Sichuan Province	247.04±24.7	205.54±21	61.93±6.3	9.96±1.02	9.96±1.02	5.73±0.58	1.15±0.1	0.29±0.03	0.03
